# Artificial intelligence in soil microbiome-driven agriculture: from practical limits to a translational roadmap

**DOI:** 10.3389/frmbi.2026.1860559

**Published:** 2026-07-03

**Authors:** Acharya Balkrishna, Priyanka Chaudhary, Shelly Singh, Anishka Saini, Aditi Kumari, Khushi Ishika Mahato, Vedpriya Arya

**Affiliations:** 1 Patanjali Herbal Research Division, Patanjali Research Foundation, Haridwar, Uttarakhand, India; 2 University of Patanjali, Haridwar, Uttarakhand, India; 3 Department of Bioscience and Biotechnology, Banasthali Vidyapith, Rajasthan, India; 4 Department of Biotechnology, Babasaheb Bhimrao Ambedkar University, Lucknow, India

**Keywords:** artificial intelligence, precision agriculture, soil microbiome, multi-omics integration, microbiome engineering, soil health, machine learning, translational agriculture

## Abstract

**Background:**

Soil microbiome research has been revolutionized by advances in high-throughput sequencing and multi-omics technologies, generating massive datasets that capture the taxonomic, functional, and metabolic diversity of microbial communities in agricultural soils; however, interpreting these complex datasets and translating them into practical agronomic insights remains challenging.

**Objectives:**

To critically assess the role of artificial intelligence (AI) in soil microbiome-driven agriculture, focusing on methodological developments, prediction performance, existing limitations, and translational opportunities.

**Methods:**

A narrative review was conducted to evaluate commonly used AI approaches, including random forest, gradient boosting, support vector machines, and deep learning architectures, alongside key microbiome data types such as amplicon sequencing, metagenomics, and functional gene profiling, with integration of environmental, agronomic, and meteorological datasets.

**Results:**

The prediction of crop productivity, disease risk, nutrient cycling dynamics, and soil health indicators may be enhanced by AI-assisted integration of microbiome, soil physicochemical, and meteorological data, according to several studies. However, broad generalizations about predictive robustness and generalizability are limited by significant diversity in datasets, validation methods, and model architectures.

**Discussion:**

To address these limitations, a five-phase implementation framework integrating centralized data systems, AI-driven analytics, multi-omics profiling, standardized soil sampling, and feedback-based model retraining within precision agriculture systems is proposed, providing a pathway for translating microbiome insights into field-scale decision support.

**Conclusion:**

AI-enabled soil microbiome applications hold significant potential for sustainable agriculture, but future advancements will require large, multisite datasets, improved validation strategies, interpretable modeling approaches, and integration with digital agriculture technologies, highlighting both opportunities and practical constraints.

## Graphical Abstract

**Figure:**
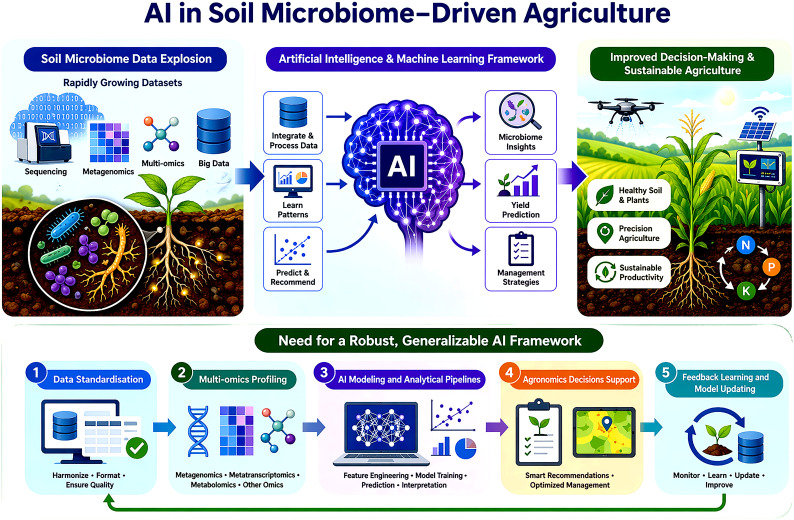

## 1 Introduction

Agriculture is undergoing a major technological transformation in response to escalating global challenges, including climate change, soil degradation, declining crop productivity, excessive agrochemical dependence, and increasing food demand. Soil deterioration and yield intensification continue to exist worldwide despite decades of agronomic progress, underscoring a crucial gap between mechanistic understanding of soil systems and their translation into predictive, field-level management tactics (Amundson et al., 2015; Balkrishna et al., 2021). Exploring the biological processes that govern soil fertility, resilience, and ecosystem stability is now essential to maintaining agricultural productivity, especially in the face of land-use intensification and climatic unpredictability (Singh et al., 2020; Pace et al., 2025). The soil microbiome, a varied community of bacteria, fungi, archaea, protozoa, and viruses, has gained much attention recently, due to its key role in nutrient cycling, plant health, and biogeochemical stability (Carswell et al., 2025). Rather than being accidental soil inhabitants, microbial communities are significant predictors of agro-ecosystem performance and are positioned as biological bases for sustainable agriculture intensification due to their various roles in maintaining soil quality (Trivedi et al., 2020). Research has demonstrated that fungal hyphae and microbial biomass support soil aggregation and structural stability, which in turn affects porosity, water infiltration, and root penetration characteristics crucial for long-term crop performance (Pandey and Saharan, 2025). Additionally, through biochemical antagonism and ecological competition, they are also shown to inhibit pathogen proliferation in the soil ecosystem (Ansabayeva et al., 2025). Innovations in systems biology, metagenomics, metabolomics, and high-throughput sequencing have shown that soil microbial communities are dynamic ecological networks that have the power to significantly impact agricultural productivity and sustainability. However, microbiological signals are extremely context-dependent, temporally dynamic, and poorly transferable across agroecological zones, making them too complex to convert into useful agronomic indicators. Techniques such as amplicon sequencing have significantly enhanced microbial taxonomic profiling; nonetheless, its capacity to resolve ecological interactions and predict functional activity is still constrained. Furthermore, comparisons between studies are made more difficult by sampling design variability, which includes variations in soil depth, spatial replication, seasonal timing, and site heterogeneity. Reproducibility and cross-study synthesis are often challenged by bioinformatics-related variabilities like differences in sequence quality control, reference database selection, and taxonomic assignment pipelines, which can also introduce inconsistencies in microbial classification and downstream ecological interpretation (Leite et al., 2022; Kumar et al., 2024). An ongoing analytical challenge is the integration of disparate information, such as microbial abundance matrices, physicochemical soil characteristics, meteorological records, and spatial metadata (Awais et al., 2023).

Artificial intelligence (AI) and machine learning (ML) have become popular methods for solving challenging computational issues in the environmental sciences and agriculture. ML methods like random forest (RF) and support vector machine (SVM) are being used more and more to forecast soil properties, microbial diversity indices, nutrient status, and crop yield (Padarian et al., 2020). Supervised modeling techniques for intricate ecological datasets have been further improved by frameworks for efficient microbiome-based categorization (Topçuoğlu et al., 2020). Simultaneously, agricultural systems have embraced statistical ML techniques to predict plant phenotypes and variability at the field level (Rehman et al., 2019). Recurrent neural networks allow temporal modeling of ecosystem responses (Phillips et al., 2020), while deep learning (DL) architectures, which can model non-linear biological relationships, are being used frequently for high-dimensional metagenomic research. The spatial integration of edaphic variables is made easier by digital soil mapping and ML-assisted soil modeling, which may enhance the interpretation of microbial dispersion patterns throughout agricultural landscapes (Porder et al., 2015). Similarly, by allowing field-scale monitoring of crop stress, vegetation dynamics, and environmental variability that affect rhizosphere microbial interactions, remote sensing platforms may supplement microbiome-based analysis (Zhang et al., 2019). The significant regional variation of soil microbial communities that predictive models must take into account across climate and land-use gradients is further demonstrated by global microbial mapping projects (Delgado-Baquerizo et al., 2018). Furthermore, by facilitating standardized, high-resolution soil sample and environmental data gathering for subsequent molecular and AI-based studies, robotics and automated sampling technologies can promote microbiome-driven agriculture (Bechar and Vigneault, 2016).

Despite these advances, the reported predictive success of AI models is often context-specific, and in many cases, improvements over traditional statistical or soil–climate models remain modest or insufficiently benchmarked. Algorithm creation and reported performance indicators have been the main focus of previous studies and reviews, while validation rigor, cross-site generalization, and ecological repeatability have received relatively little attention. Concerns about ecological overfitting and exaggerated predictive performance estimates are raised by the fact that model evaluation in many published studies mostly relies on internal cross-validation rather than independent external datasets. Furthermore, it is challenging to ascertain the precise incremental predictive value contributed by microbiome-derived features because comparatively few studies systematically compare microbiome-informed models against abiotic-only models based only on soil physicochemical or climatic variables. As a result, it is yet unclear if microbiome-informed AI models offer truly unique prognostic insights or if they mostly replicate environmental cues already detected by traditional predictors. Therefore, there is still a significant gap between experimental innovation and scalable agricultural adoption, even if AI-driven soil microbiome research holds great promise. To address these gaps, in addition to technology advancements, translational frameworks that incorporate biological relevance, economic viability, infrastructural accessibility, regulatory concerns, and farmer-centric implementation techniques must be developed. In this review, we critically analyze the current applications of AI in soil microbiome-driven agriculture, emphasizing both its practical constraints and its transformative potential. Additionally, through scalable, comprehensible, and financially feasible AI-enabled agricultural systems, we offer a translational roadmap that aims to connect laboratory-scale discoveries with field-level application. So the review aims to examine:

- AI techniques used in soil microbiome datasets, focusing on data architecture, algorithm choice, and the combination of environmental and multi-omics factors.
- The empirical data for cross-site generalizability, validation rigor, and predictive gain in order to ascertain whether AI significantly improves agronomic forecasting over traditional statistical methods.
- The methodological, ecological, and translational constraints that offer a roadmap for future AI-driven microbiome applications in sustainable agriculture that are scalable, interpretable, and in line with field-level implementation.

## 2 Methodology

A structured narrative review framework was used to ensure thorough, impartial, and critically interpretive coverage of the existing literature due to the constantly growing and extremely interdisciplinary nature of AI-enabled soil microbiome research. In addition to assessing the translational viability and practical constraints related to real-world implementation, the review was intended to synthesize emerging evidence at the nexus of soil microbial ecology, computational biology, precision agriculture, environmental informatics, and machine learning-driven agricultural decision systems. Major scientific databases, such as Scopus, Web of Science, PubMed, and Google Scholar, were used to do an extensive literature search in order to capture the development of next-generation sequencing technology, advancements in agricultural microbiome analytics, and the recent incorporation of AI and machine learning techniques into soil and crop management systems. Through manual reference list screening, citation chaining, and analysis of highly cited review articles and fundamental methodological studies in the field, more pertinent papers were located.

Using phrase combinations and Boolean operators related to AI technologies, microbiome analytics, and agricultural applications, search queries were created iteratively. “Artificial intelligence” AND “soil microbiome,” “machine learning” AND “rhizosphere,” “deep learning” AND “soil microbial communities,” “AI-driven agriculture” AND “metagenomics,” “precision agriculture” AND “microbial ecology,” “soil health prediction” AND “machine learning,” and “microbiome-informed decision systems” AND “sustainable agriculture” were some examples of search terms. Studies that addressed agricultural outcomes like crop productivity, nutrient cycling, soil health, stress detection, or biofertilizer design were taken into consideration for inclusion if they i) used soil or rhizosphere microbiome datasets; and ii) applied AI, machine learning, deep learning, or computational predictive approaches. Peer-reviewed journal articles that provided methodological information, validation techniques, or integrative microbiome-environmental investigations were given preference.

The main comparison analysis did not include non-review papers, conference abstracts, and studies that lacked methodological description, soil microbiome applications, or relevance to agriculture. The AI approaches covered in this study were found through narrative synthesis of frequently described methodologies across the chosen literature. To illustrate significant thematic advancements, such as integrated smart-agriculture systems, stress-response prediction, microbiome-assisted nutrient management, and predictive crop modeling, recent studies from 2020–2025 were included in case studies.

## 3 Soil microbiome: biological foundations

One of the most varied biological systems on Earth is the soil microbiome, which is made up of bacteria, fungi, viruses, protists, and archaea. Microbial diversity varies significantly among soil types, climatic zones, and land-use systems. Abiotic elements like pH, moisture, texture, and nutrient availability, as well as biotic interactions like plant roots and microbial competition, affect the composition of the soil microbial community (Ahammed et al., 2025; Iqbal et al., 2025). While protistan abundance and total microbial biomass are substantially influenced by moisture and texture, soil pH is often identified as a primary predictor of bacterial community structure over broad regional scales. However, microbiome composition is not universally determined by a single environmental condition, leading to significant variation among soil strata and ecosystems. While specialized metabolic functions may rely on more specific taxonomic groups, broad ecological processes like organic matter breakdown are frequently supported by functionally redundant microbial taxa (Chen et al., 2022). Organic matter decomposition, carbon sequestration, nutrient cycling (such as nitrogen fixation and phosphorus solubilization), soil aggregation, and pathogen suppression are just a few of the vital ecosystem processes that soil microbiomes support. Numerous studies have also connected increased microbial diversity to enhanced respiration, enzymatic activity, and agricultural productivity (Hartmann and Six, 2023; Romero et al., 2025; Balkrishna et al., 2026). Plant growth-promoting rhizobacteria (PGPR), mycorrhizal fungi, and nitrogen-fixing microbes are examples of rhizosphere-associated microorganisms that can improve plant nutrient uptake, growth, stress tolerance, and disease resistance through processes involving root exudates, phytohormones, and extracellular enzymes. Environmental factors and management techniques have a significant impact on these dynamic plant–microbe interactions (Huang et al., 2021; Kumar et al., 2023; Zaman, 2025; Panek et al., 2026).

However, it has been observed that both culture-dependent and sequencing-based constraints continue to limit our current understanding of soil microbial diversity. Experimental assessment of the ecological roles of soil microorganisms is limited since many of them are still uncultivable under typical laboratory settings. Also, relic DNA from non-viable species may inflate sequencing-based diversity estimates, which could have an impact on comparative analyses and ecological interpretation (Chen et al., 2022; Ansabayeva et al., 2025; Basset and Zaldo-Aubanell, 2025). Many taxa require extremely particular growth circumstances, metabolic requirements, or symbiotic interactions that are challenging to replicate under typical laboratory conditions. As a result, culture-based techniques frequently induce representation bias by underrepresenting species with slower growth rates or specific ecological requirements while favoring recovering microorganisms that easily grow under the chosen incubation and media conditions. Furthermore, without supplementary genomic or metabolomic investigations, traditional methods like plate counts offer little functional information. While proteomic analyses often require large biomass inputs that can limit scalability, sequencing-based methods may also be impacted by relic DNA derived from non-viable cells, potentially inflating diversity estimates (Sergaki et al., 2018; Gupta et al., 2022; Awais et al., 2023; Pace et al., 2025). Microbiome data quality and recovery efficiency are also influenced by the physicochemical characteristics of the soil. Incomplete or uneven DNA recovery and possible biases in microbial community profiling, for instance, can result from clay particles and humic substances interfering with cell dislodgement, DNA extraction, and downstream amplification processes (Stefani et al., 2015; Gupta et al., 2022; Kapinusova et al., 2023). Results get biased when soil pH, moisture, texture, and nutrients are ignored since these factors influence community changes but are rarely assessed in conjunction with cultures, making reproducibility and meta-analyses more difficult. Snapshots from single samplings are further distorted by temporal and spatial variability, such as seasonal variations and unequal microbial distribution (Geisen, 2021; Leite et al., 2022).

## 4 Multi-omics approaches in soil microbiome

Due to the complexity associated with soil microbiome assessment methods, research on soil microbiomes has shifted from descriptive taxonomy to multilayered molecular profiling due to developments in high-throughput sequencing and metagenomic technologies. These methods produce a variety of data types with varying biological interpretations, dimensionalities, and structures. Assessing predictive modeling techniques in soil systems requires an understanding of these data levels.

### 4.1 Need for multi-omics approaches

Multi-omics approaches integrate complementary molecular datasets to provide a more comprehensive understanding of soil microbial communities, their functions, and their interactions with plants and environmental conditions. Different omics layers capture distinct biological information rather than equivalent signals. For example, amplicon sequencing primarily provides taxonomic composition based on marker genes, whereas metagenomics characterizes the broader genetic potential of microbial communities. Furthermore, metatranscriptomics evaluates actively expressed genes, metaproteomics assesses translated proteins and enzymatic functions, and metabolomics profiles metabolites associated with microbial and plant biochemical activity. Therefore, ecological interpretation may be limited by relying on a single omics layer. For example, transcriptomic or metabolomic profiles may more accurately reflect microbial activity and stress responses in real time, whereas metagenomic datasets may discover functional genes without verifying whether these genes are actively expressed under field settings. In heterogeneous soil environments, where local environmental factors have a significant impact on microbial functional activity, this distinction is especially crucial (Kimotho and Maina, 2024; Degnan et al., 2025). Multi-omics frameworks can enhance the identification of microbial consortia, functional biomarkers, and metabolic pathways linked to soil health, plant production, and stress resilience by combining metagenomics, metatranscriptomics, metaproteomics, and metabolomics. Through feature selection and cross-layer validation techniques, these integrative approaches may also lower data dimensionality and enhance signal detection; nevertheless, their efficacy is largely dependent on study design, computational pipelines, and data harmonization techniques. Multi-omics approaches are increasingly being investigated to better understand rhizosphere mechanisms, microbial interactions, and sustainable soil management strategies under changing environmental conditions, despite persistent challenges related to standardization, computational complexity, and interoperability (Arıkan and Muth, 2023).

### 4.2 Types of multi-omics data

According to Aguiar-Pulido et al. (2016), “metagenomics, metatranscriptomics, metaproteomics, and metabolomics represent complementary multi-omics approaches used in soil microbiome research to investigate microbial composition, functional potential, activity, and biochemical interactions beyond the limitations of single-method analyses.” The main use of amplicon-based sequencing techniques, such as 16S rRNA or ITS marker-gene sequencing, is metataxonomic profiling, which describes the relative taxonomic diversity and composition of microbial communities. Shotgun metagenomics, on the other hand, analyzes all ambient DNA to evaluate the functional potential and wider genetic repertoire of microbial communities, including genes related to stress responses and nutrient cycling (Shaffer et al., 2022). However, rather than directly identifying biological activity, metagenomic techniques typically infer possible functioning. In order to separate metabolically active microorganisms from dormant populations, metatranscriptomics expands this analysis by sequencing community RNA to identify actively expressed genes and dynamic microbial responses under environmental conditions like drought or salinity stress (Zhang et al., 2019). Although soil matrix complexity and low protein recovery can limit analytical throughput, metaproteomics further assesses translated proteins and enzymatic machinery using mass spectrometry-based techniques, providing more direct evidence of functional processes like organic matter decomposition and nutrient transformation. In order to connect microbial activity with soil biochemical processes and plant signaling pathways, such as the synthesis of osmoprotectants, exudates, and antimicrobial compounds, metabolomics profiles small molecules and metabolic intermediates; liquid chromatography–mass spectrometry (LC-MS) or related analytical platforms are frequently used (Aguiar-Pulido et al., 2016).

### 4.3 Challenges in multi-omics data

Multi-omics datasets suffer from high dimensionality because high-throughput sequencing produces enormous amounts of DNA fragments per soil sample in addition to transcriptomic, proteomic, and metabolomic layers. This results in massive, complex databases that exceed processing, storage, and interpretation capabilities (Quince et al., 2017; Knight et al., 2018). Sequence data from marker genes, such as 16S rRNA for bacteria and fungal internal transcribed spacer for fungi, exhibit intrinsic compositional sparsity, with feature counts restricted to relative abundances rather than absolute values, and integrating different streams, such as metagenomics with environmental metadata, necessitates error modeling over arbitrary clustering for biological resolution (Fasolo et al., 2024).

Analysis is made more difficult by spatial and temporal variability, which obscures consistent signals across agroecological zones because soil microbial communities vary greatly due to climate, crop types, land-use history, temperature, precipitation, pH, and soil characteristics (Fierer, 2017; Bahram et al., 2018). These elements contribute to noise and biases, as do methodological flaws like the inability of traditional bioinformatics to capture non-linear interactions or intricate networks of microbial cooperation, competition, and metabolic exchange; subjective trait identification; and practical timeframes for multistream integration (Faust and Raes, 2012). High dimensionality, data heterogeneity, geographical and temporal variability, and widespread noise and biases present significant hurdles for multi-omics data analysis in soil microbiome research, requiring sophisticated AI integration for significant discoveries.

## 5 AI methodologies in soil microbiome research

By enabling machines to carry out tasks that need human-like intellect, AI is being employed more and more in agriculture to increase agricultural output, efficiency, and sustainability. Furthermore, modern agricultural methods have been greatly enhanced by the incorporation of technologies like the Internet of Things (IoT), decision support systems (DSSs), cloud computing, big data analytics, and remote sensing (Paul et al., 2026).

A subfield of AI called ML creates algorithms to learn from data and carry out particular tasks. By fitting models to input–output pairs, supervised learning employs labeled data to predict outputs, which can be either numerical values (regression) or categories (classification). After that, these models can generalize to fresh data, although overfitting must be avoided. On the other hand, unsupervised learning finds patterns in unlabeled data. It is helpful for data exploration and enhancing feature selection for supervised learning, even though its performance is more difficult to assess. Because ML makes it possible to analyze complicated, high-dimensional biological data, it plays a significant role in soil microbiome research. It aids in finding trends and connections between microbial communities and environmental elements like plant health, soil characteristics, and climate. Based on microbial composition and physicochemical factors, ML models in supervised learning can forecast outcomes such as agricultural production, disease occurrence, or soil fertility. Microbial communities can be clustered, diversity patterns can be found, and important functional groupings can be identified without prior labeling using ML in unsupervised learning.

Because soil microbiome datasets are frequently sparse, non-linear, heterogeneous, and highly dimensional, ML integration is very pertinent. For instance, each sample in a metagenomic dataset may have millions of sequence-derived characteristics, requiring computational methods for biomarker identification, functional annotation, taxonomic categorization, and modeling intricate environmental interactions. However, there are a number of domain-specific methodological difficulties when using ML in soil microbiome systems. Because microbiome datasets are compositional by nature, relative abundances are interdependent and, if studied without proper normalization or modification, may result in false relationships. Statistical modeling and feature interpretation are made more difficult by zero inflation brought on by uncommon or undiscovered taxa. Additionally, significant technical heterogeneity across studies might be introduced by batch effects resulting from variations in sequencing platforms, DNA extraction techniques, sampling strategies, and bioinformatics pipelines. Strongly associated soil physicochemical and meteorological variables can also complicate predictive interpretation, making it challenging to separate the independent contribution of microbiome-derived traits. Data leakage across training and testing datasets is another serious issue, especially when chronologically or geographically related samples are inadvertently included across validation partitions. This could inflate estimates of predictive performance and decrease apparent generalizability. **Table 1** summarizes the main AI methods currently used in soil microbiome-based agricultural prediction and compares them based on input data type, application domain, methodological strengths, and significant limitations.

**Table 1:** AI techniques used in soil microbiome research: data sources, uses, and methodological issues.

Method category	AI/analytical method	Learning type	Primary data inputs	Primary application	Major strengths	Major limitations	Representative reference
Ensemble machine learning	Random forest (RF)	Supervised ML	OTU/ASV abundance tables, soil physicochemical variables, environmental metadata	Soil health classification, nutrient prediction, crop response modeling	Handles non-linear relationships; robust to noisy and high-dimensional data; supports feature ranking	Limited biological interpretability; feature importance may be biased by correlated predictors and compositionality; risk of overfitting with small datasets	Wilhelm et al. (2022), Dutta et al. (2022)
Ensemble machine learning	Gradient boosting/XGBoost	Supervised ML	Microbiome profiles, climatic and agronomic variables	Yield prediction, nutrient forecasting, disease-risk prediction	High predictive performance; effective handling of heterogeneous datasets	Sensitive to hyperparameter tuning; may require extensive optimization and large datasets	Afzal et al. (2025)
Exploratory ordination and unsupervised analysis	Principal component analysis (PCA), clustering approaches	Unsupervised analysis/dimensionality reduction	OTU/ASV abundance matrices and environmental variables	Community structure exploration, ecological pattern identification, visualization	Summarizes multivariate ecological patterns; useful for exploratory interpretation	Not inherently predictive; sensitive to scaling and preprocessing choices	Shi et al. (2022), Jackson et al. (2017)
Deep learning	Transformer architectures	Deep learning	Multi-omics datasets and environmental metadata	Integrated crop-response forecasting, complex feature interaction modeling	Captures non-linear multilayer interactions; supports attention-based modeling	Computationally intensive; limited external validation in agricultural microbiome studies	Adhikary et al. (2025)

PCA and clustering approaches are included as exploratory or dimensionality-reduction techniques and should not be interpreted as equivalent to predictive AI models. Learning-type classification indicates whether the approach is primarily supervised machine learning, unsupervised analysis, deep learning, or integrative AI-based modeling.

OTU, operational taxonomic unit; ASV, amplicon sequence variant; RF, random forest.

## 6 AI applications in microbiome-driven agriculture

AI applications for soil health evaluation, disease risk prediction, crop yield forecasting, nutrient cycling analysis, and microbiome-informed biofertilizer development are being investigated largely in microbiome-driven agriculture. Instead of completely proven large-scale agricultural use, current studies mostly show predicted associations and experimental or pilot-scale applications. In order to support adaptive soil-management strategies, soil health prediction frameworks often incorporate multi-omics, sensor, and environmental datasets using ML techniques like random forest to analyze variables like pH, organic carbon, microbial diversity, and nutrient status. Deep learning and metagenomic profiling have also been used in nutrient availability modeling to study microbial processes related to phosphate solubilization, nitrogen fixation, and micronutrient cycling by taxa such as plant PGPR. Although field-scale reproducibility and long-term validation are still limited, some research indicates that microbiome-informed nutrient recommendations may lessen reliance on synthetic fertilizers in controlled or experimental conditions (Pandey and Saharan, 2025; Balkrishna et al., 2026). Similarly, to enhance predictive performance for crops like maize and sorghum, crop-yield prediction studies have combined microbiome characteristics with unmanned aerial vehicle (UAV) imagery and environmental metadata using gradient boosting and related ML frameworks (Logeshwaran et al., 2024; Schaks et al., 2025). Predictive accuracy, however, varies significantly according to validation methodology, environmental heterogeneity, and dataset makeup. Similarly, through optimal microbial consortia selection, AI-guided microbiome-based fertilizer design has demonstrated promising experimental results for nutrient uptake and residue breakdown; nevertheless, large-scale translational validation and commercial deployment are still in their infancy (Xie et al., 2025).

### 6.1 Case studies and recent advancements (2020−2025)

AI-enabled soil microbiome research has rapidly expanded from proof-of-concept studies to more integrated and field-relevant applications, according to representative peer-reviewed case studies published between 2020 and 2025. Three main developments are demonstrated by recent case studies: i) integrative yield prediction models, ii) the discovery of microbial features for the design of biofertilizer and nutrient cycling, and iii) the confluence of microbiome analytics with smart agricultural technologies. Rather than offering a comprehensive systematic inventory, the case studies compiled in **Table 2** were narratively synthesized to demonstrate significant methodological and application trends documented throughout this time. External validation and cross-regional transferability are still underreported, despite the fact that predictive performance has generally increased. Furthermore, rather than serving as major indicators, microbiome traits often serve as complementary ones. These trends imply that multisource ecological modeling frameworks yield the greatest AI-driven advances, highlighting the significance of integrated data architectures for reliable agricultural forecasts.

**Table 2:** AI applications in soil microbiome-based agricultural prediction: case studies (2020–2025).

Study	Target outcome	Data inputs	AI methods used	Integration level	Validation strategy	Reported performance	Key contribution	Major limitations
Logeshwaran et al. (2024)	Maize yield prediction	Soil moisture, temperature, fertilizer input	Deep learning framework	Environmental and agronomic (limited microbiome emphasis)	Internal validation	High predictive accuracy; rapid processing	Demonstrated speed and scalability of AI in yield estimation	Limited biological interpretability; unclear cross-site generalization
Schaks et al. (2025)	Maize yield variability	Microbial taxa (*Hyphomicrobium*, *Aeromicrobium*) and regional soil data	Machine learning regression	Microbiome and soil context	Field-level validation	Explained 65% variance in yield	Linked specific microbial groups to yield performance	Region-specific; uncertain external transferability
Aghdam et al. (2024)	Potato yield and disease susceptibility	Microbiome profiles, soil physicochemical and climate variables	Supervised ML models	Multilayer integration	Internal cross-validation	Improved accuracy vs. abiotic-only models	Showed microbiome enhances prediction when integrated	Microbiome-alone predictive power limited
Shariati and Abbasi (2025)	Winter wheat yield (national scale)	Microbiome data, soil moisture, vegetation indices and climate	eXtreme Gradient Boosting model	Multisource (microbiome, remote sensing, and climate)	Multiregion dataset	High country-level predictive accuracy	Demonstrated multisource AI forecasting	Dependent on large, harmonized datasets
Asad et al. (2023)	Wheat grain quality	Microbial diversity indices + nitrogen-cycle gene abundance	Least Absolute Shrinkage and Selection Operator (LASSO) regression	Biological and functional gene integration	Seasonal dataset	Strong early-season predictive capacity	Showed biological data; predict crop quality	Limited to a specific crop and region
Li and Li (2025)	Regional nitrogen cycling dynamics	Cropland microbial composition and nitrogen input data	ML-based synthesis	Regional ecological integration	Large-scale dataset analysis	Identified relationships between N inputs and nutrient turnover	Linked microbiome patterns to nutrient cycling	Correlative rather than mechanistic
Hagen et al. (2024)	Drought stress classification	16S rRNA relative abundance	RF classifier	Microbiome-only	Classification accuracy testing	High stress classification accuracy	Identified microbial markers responsive to environmental stress	Indirect link to nutrient cycling; function inferred
Díaz-Rodríguez et al. (2025)	Biofertilizer design	Large soil microbiome datasets and functional traits	ML-based microbial selection	Functional and ecological integration	Experimental validation phase	Identified microbial consortia enhancing N and P dynamics	Transition from prediction to intervention	Still early-stage translational validation
Sørensen et al. (2025)	Crop health	Fungal OTU abundance profiles	Random forest (RF)	Remote sensing, soil microbiome	Internal train–test split, repeated cross-validation	RF models achieved higher predictive performance	Demonstrated integration of satellite-derived vegetation indices	Limited biological sample size
Singh et al. (2025)	Beneficial microbial consortium prediction	Microbial interaction datasets	Machine learning screening	Microbial interaction modeling	Computational validation	Predicted synergistic microbial combinations	Advanced rational biofertilizer design	Requires field-scale confirmation
Pandey and Saharan (2025)	Integrated soil–crop ecosystem simulation	Microbiome data and Internet-of-things sensors and UAV imagery	AI-integrated digital twin	Fully integrated smart agriculture	System-level testing	Real-time soil ecosystem modeling	Demonstrated AI, microbiome and Internet-of-things convergence	Data harmonization and scalability challenges

## 7 Promise and performance of AI models: validation and generalizability

Despite numerous research reporting increased prediction performance after integrating microbiome variables with soil physicochemical and meteorological parameters, direct head-to-head benchmarking against abiotic-only models is still very limited. It is challenging to separate the independent predictive contribution of microbiome-derived characteristics in many published frameworks since microbial, environmental, and agronomic variables are all included at the same time. Additionally, rather than using geographically independent external datasets, a number of research mainly rely on internal cross-validation, which could inflate claimed performance estimates and lower trust in cross-site generalizability. Therefore, rather than being a consistently better stand-alone predictor, current research indicates that microbiome-informed AI works best as a complementing predictive layer linked with environmental variables.

### 7.1 Validation and generalization in microbiome-based yield prediction

Predictive success is highly dependent on the integration of microbiome data with environmental and physicochemical characteristics, as well as the rigor of validation techniques used, according to benchmark evaluations of agricultural and soil microbiome AI investigations. The type of evaluation framework described in each study was used to compare the validation rigor in this review. Techniques like train-test partitioning and k-fold cross-validation carried out within the same dataset are referred to as internal validation. Reserving a portion of samples for testing without repeatedly resampling is known as hold-out validation. Separate inner and outer loops are used in nested cross-validation for performance estimation and hyperparameter tweaking. While external or cross-study validation tests performance using entirely independent datasets not used in model training, leave-one-site-out validation assesses model transferability across geographically varied sampling locations. Furthermore, cross-region testing assesses generalizability across various agroecological zones, and temporal validation looks at predicted robustness across several sampling periods or crop seasons. The most commonly used models in the chosen research were random forest-based architectures, which were followed by gradient boosting, ensemble learning frameworks, Bayesian neural networks, and spatiotemporal deep learning techniques. Only a small number of research used independent external or cross-study validation frameworks, whereas the majority used train-test partitioning or k-fold cross-validation.

Microbiome-informed AI models show significantly varying prediction accuracy depending on dataset structure, preprocessing approach, taxonomic resolution, and validation rigor, according to comparative benchmarking studies. Barbosa et al. (2025) showed that microbiome-based prediction frameworks had moderate cross-study transferability, outperforming four cutting-edge benchmarking tools on 21 out of 24 separate datasets and attaining a mean absolute percentage error of approximately 11% during external validation. However, compositional sparsity and preprocessing variability continued to have a significant impact on predictive stability. In the same way, Aouabed et al. (2026) found that while predictive accuracy significantly decreased at finer taxonomic scales, bacterial phylum-level models attained a maximum *R*^2^ of roughly 0.57, underscoring the increased risk of sparse-feature instability and ecological overfitting in highly resolved microbiome datasets. The study’s fungal functional prediction models performed only mediocrely (*R*^2^ = 0.45), highlighting the difficulties in predicting the microbiome in various environmental settings.

In general, integrated prediction frameworks perform better than microbiome-only methods as highlighted through the studies. For instance, Aghdam et al. (2024) have shown that, in comparison to microbiome-only predictors, combining soil physicochemical characteristics and microbial-density factors with microbiome profiles significantly increased prediction accuracy for potato yield and disease susceptibility. Crucially, the study also showed that the machine-learning algorithm itself frequently had less of an impact on model performance than preprocessing decisions, feature-selection techniques, and label definition. Studies on agricultural forecast that exhibit remarkably high internal validation accuracy without independent external benchmarking raise similar issues. For example, Afzal et al. (2025) used an ensemble RF-XGBoost framework trained on soil nutrients and climatic variables to achieve approximately 98% crop recommendation accuracy; however, the study mainly relied on internal cross-validation and lacked geographically independent testing and microbiome integration. Similarly, Gao et al. (2025) showed that LSTM architectures and spatiotemporal graph neural networks enhanced the forecasting of longitudinal microbiome dynamics when compared to isolated modeling approaches. However, validation was mainly limited to control longitudinal datasets rather than heterogeneous field-scale agroecosystems.

### 7.2 Interpretability and biological insight

Even beyond predictive performance, interpretability is essential for agronomic adoption since AI-driven forecasts must eventually be converted into biologically significant theories and practical farm management choices. Supervised machine learning algorithms often give feature-importance rankings that can direct ecological interpretation and management methods, in addition to discovering microbial taxa or functional genes linked to yield, nutrient cycle, or disease resistance. The identification of advantageous microbial consortia with AI assistance demonstrates this translational potential. For instance, the rational design of next-generation biofertilizers has been made easier by the application of machine learning techniques to discover microbial assemblages linked to enhanced plant growth, nutrient availability, and stress resilience (Pace et al., 2025). Similar to this, AI-assisted ecological modeling has made it possible to identify functionally significant microbial networks associated with increased agricultural productivity and ecosystem resilience by revealing synergistic microbial interactions that might go unnoticed through traditional analytical methods (Rahman and Das, 2025; Delgado-Baquerizo et al., 2018). In order to enhance biological interpretability and identify microbial drivers linked to agronomic traits, explainable artificial intelligence (XAI) techniques like random forest feature-importance ranking, Least Absolute Shrinkage and Selection Operator (LASSO)-based variable selection, SHapley Additive exPlanations (SHAP), partial dependence plots, attention-based deep learning architectures, and ecological network analysis have also been increasingly used in new age studies (Topçuoğlu et al., 2020; Pasolli et al., 2016). By directly connecting prediction outcomes with nutrient cycling pathways, enzyme activity, or microbial metabolic functions rather than only depending on taxonomic abundance profiles, functional-gene-based machine learning frameworks further enhance interpretability. Despite these developments, many ML models continue to function as “black boxes,” making it challenging to comprehend the ecological principles underpinning their predictions, rendering interpretability a significant obstacle in AI-driven soil microbiome research (Pandey and Saharan, 2025). This restriction is especially noticeable in deep learning and complicated ensemble structures, where high prediction accuracy does not always translate into biologically significant inference. Feature-importance rankings may not correctly reflect true ecological function or causal biological links due to the varied, sparse, compositional, and highly correlated nature of microbiome datasets. According to Topçuoğlu et al. (2020), preprocessing techniques and feature-selection methods have a significant impact on microbiome classification models, raising the possibility of erratic or deceptive interpretations. Similar to this, Vázquez-Baeza et al. (2018) pointed out that batch effects, sequencing biases, and confounding environmental variables can produce misleading predictive associations that seem statistically significant but lack ecological validity. As a result, mechanistically significant microbial pathways or agronomically significant functions are not always associated with statistically significant predictions.

The uncertainty surrounding the independent predictive significance of microbiome-derived factors is another significant obstacle. Microbial variables primarily served as secondary or context-dependent predictors, whereas environmental covariates like soil pH, nutrient availability, climate, or moisture drove improvements in prediction accuracy as shown in a number of studies (Pace et al., 2025; Hagen et al., 2024; Geisen, 2021; Leite et al., 2022). This draws attention to a crucial difference between mechanical ecological comprehension and correlation-driven prediction, where AI models may be highly predictive without comprehending biologically causal linkages. Further complicating feature selection and raising the likelihood of spurious correlations are sequencing-depth variability, multicollinearity, zero inflation, and sparse, high-dimensional feature matrices, especially when datasets are still small or geographically limited.

Future microbiome-AI research should use standardized interpretability and reporting frameworks that clearly document the following: i) assumptions about compositional microbiome data structure, ii) preprocessing and normalization procedures, iii) feature-selection and dimensionality-reduction strategies, iv) treatment of sparse and zero-inflated variables, v) uncertainty estimates and confidence intervals associated with predictions, vi) external validation across sites and seasons, and vii) biological justification for selected predictive features. When converting AI conclusions into agricultural advice or microbiome-engineering methods, it is crucial to make a clear distinction between purely statistical connections and mechanistically supported ecological reasoning. Therefore, combining explainable AI with ecological theory, functional multi-omics profiling, and independent field validation may pave the way for more reliable, comprehensible, and biologically based decision support systems that can connect computational predictions with practical agronomic interventions and sustainable soil-management techniques.

### 7.3 Integration with smart agricultural systems

Algorithm performance is only one aspect of interpretability and validation, including system-level integration. AI-based microbiome evaluation is increasingly being integrated with digital twin simulations, IoT sensor networks, and UAV-based remote sensing platforms within larger digital agriculture ecosystems to improve predictive performance and real-time monitoring capacity. Soil temperature, moisture, pH, and nutrient availability are all continuously measured by IoT-enabled soil sensor arrays, allowing for the dynamic integration of environmental and microbiological metrics for adaptive agriculture management (Miller et al., 2025). Concurrently, high-resolution field-scale monitoring of crop physiological features, vegetation indices, canopy stress responses, and spatial heterogeneity is supported by UAV platforms fitted with RGB, multispectral, hyperspectral, or thermal imaging systems. In order to investigate relationships between microbial community composition, rhizosphere processes, and crop health indices, the aboveground imaging datasets can subsequently be computationally merged with belowground microbiome profiles and soil physicochemical characteristics (Agrawal and Arafat, 2024). In order to simulate soil–plant–microbe interactions and support agricultural management decisions pertaining to irrigation, fertilization, and harvesting, digital twin frameworks are also being investigated for integrating real-time environmental sensor inputs with AI-assisted soil, crop, and microbial datasets (Miller et al., 2025). The convergence of nanotechnology, biosensing platforms, and AI-driven analytics may further strengthen smart agriculture systems by improving disease surveillance, environmental pathogen control, and sustainable soil health management as predicted through nanobased studies (Chaudhary et al., 2026). However, presently, there is a little large-scale field deployment incorporating real-time microbiome analytics into operational farm decision systems, and the majority of current applications are still in the experimental or pilot-study stage. Additionally, although AI-driven soil microbiome analytics are increasingly being integrated with smart agriculture systems, a number of constraints hinder their scalability and practical application. Smallholder or resource-constrained farming systems might not be able to afford the significant financial commitment, technical infrastructure, and upkeep needed for the deployment of IoT sensor networks, UAV-based photography, and digital twin platforms. Due to problems with data standardization, interoperability, and real-time synchronization, data integration across heterogeneous sources such as microbiome profiles, environmental sensors, and remote sensing platforms remains technically difficult. Furthermore, ambient noise, data gaps, and sensor errors can spread through AI models, decreasing system robustness and forecast accuracy. Long-term stability under dynamic field circumstances, model validation, and calibration are all made more difficult by the complexity of these linked systems. Through studies, it is also observed that increasing model complexity does not always translate into better predictive performance or generalizability. Even though transformer-based architectures and deep learning can model intricate non-linear relationships, their benefits over relatively simpler ensemble-learning techniques like random forest or gradient boosting are frequently uneven, especially when used with small, sparse, or noisy microbiome datasets.

## 8 Challenges and limitations

### 8.1 High data dimensionality

Thousands to millions of sequence-derived characteristics, such as microbial taxa, genes, transcripts, metabolites, and functional annotations across vast sample collections, can be found in soil microbiome datasets, producing extremely sparse and high-dimensional data structures. For drought stress or yield prediction, for example, microbiome-only random forest classifiers trained on local 16S rRNA abundance profiles often achieved high within-site classification accuracy; however, their predictive stability significantly declined under varying soil textures, climatic conditions, and management regimes (Hagen et al., 2024; Schaks et al., 2025). Furthermore, it is challenging to discern biologically significant trends from statistical aberrations due to the compositional and sparse nature of microbiome data, which increases noise-to-signal ratios. Aghdam et al. (2024) found that whereas microbiome-only predictors showed poor independent generalizability, microbiome-derived variables enhanced prediction performance only when integrated with physicochemical and climatic factors. Together, the results imply that sparse taxonomic matrices with thousands of low-abundance or zero-inflated features may increase the complexity of the model and make it more vulnerable to ecological overfitting, especially when sample sizes are still relatively small. High-dimensional microbiome datasets raise the possibility of choosing bogus predictors, in which statistically significant features could not match taxa that are functionally or ecologically significant. Due to the increased dimensionality of multi-omics and environmental information, which makes feature selection, interpretation, and accurate prediction more challenging, this poses a significant restriction.

### 8.2 Danger of overfitting

**Figure 1:**
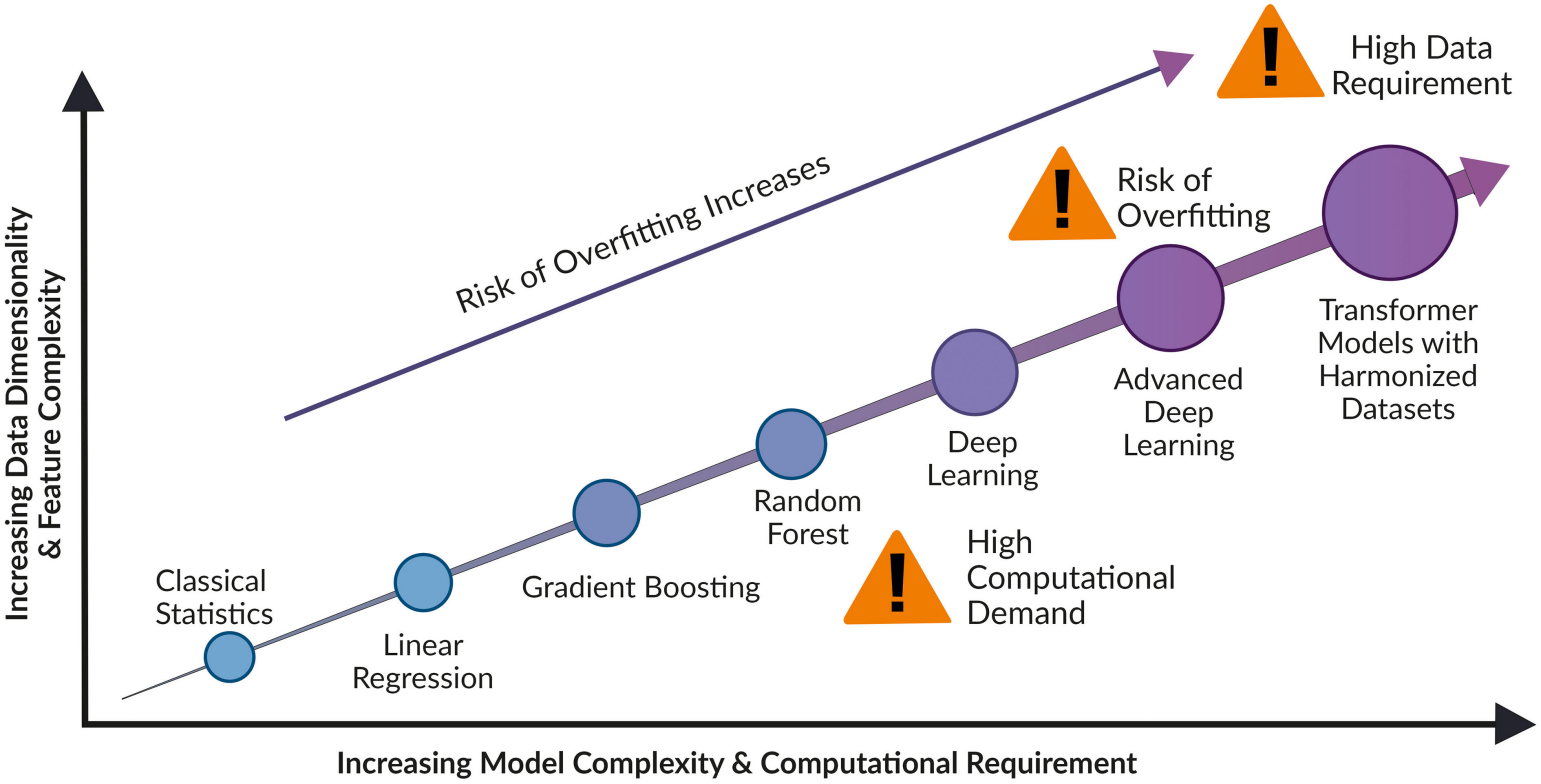
A conceptual representation of the connection between rising microbiome data dimensionality and model complexity, causing possible overfitting risk in soil microbiome analytics.

Intricate AI models that have been developed on sparse datasets might demonstrate poor global adaptability but excellent internal precision. According to Pasolli et al. (2016), because microbiome machine-learning methods collect cohort-specific microbial signatures rather than conserved biological patterns, they frequently show decreased repeatability across independent cohorts. Ecological overfitting, in which algorithms learn site-specific microbial signatures that are unable to generalize across agroecological regions, exacerbates this issue. This implies that sophisticated AI techniques may match noise or site-specific patterns rather than actual ecological correlations in soil microbiome research, particularly when datasets are limited and irregular or lack external validation. Microbiome classification models are extremely sensitive to feature sparsity and preprocessing decisions, which raises the possibility of unstable predictions and overfitting (Topçuoğlu et al., 2020). Increasing dataset dimensionality and feature sparsity may increase the danger of overfitting in microbiome prediction models, as **Figure 1** conceptually shows, especially when sample sizes are still constrained in relation to the number of predictors. However, the feature-to-sample ratio, regularization techniques, preprocessing choices, signal strength, and validation design all have an impact on overfitting risk in addition to model complexity.

### 8.3 Cost of sequencing and computational requirements

Widespread agricultural implementation is limited by the high cost and computing demands of advanced multi-omics methods. Although high-resolution techniques like shotgun metagenomics, metatranscriptomics, and multi-omics integration frequently offer deeper functional insights and may increase predictive accuracy, their large data volumes, sophisticated computational requirements, and high sequencing costs may restrict their applicability at a farm scale. Furthermore, large-scale metagenomic sequencing produces computationally demanding datasets that require substantial preprocessing, assembly, and annotation, as well as high-performance computing infrastructure, as noted by Quince et al. (2017). It has been observed that by identifying significant variables and model decision patterns, explainable AI techniques may increase transparency; nonetheless, biological validity and agronomic dependability are not ensured by model interpretability alone. Ecological confirmation and experimental validation are still needed for biological interpretation of AI-derived relationships. According to Knight et al. (2018), this exponential rise of sequencing data and metadata complexity has made microbiome studies more reliant on harmonized analytical methods and scalable computational pipelines. However, AI systems, IoT infrastructure, and sequencing all need hefty financial outlays. Unless cost-effective deployment methods are created, AI-enabled diagnostics run the risk of favoring large farms.

### 8.4 Site-specific variability

Local environmental factors, such as soil pH, texture, moisture, climate, vegetation, land-use history, and agricultural management practices, have a significant impact on the composition and function of microbial communities. Significant geographical variation in soil microbial assemblages across biomes and agroecosystems has been shown by extensive ecological research, suggesting that microbial patterns found in one place might not be transferable to another (Fierer, 2017; Delgado-Baquerizo et al., 2018; Balkrishna et al., 2026), causing site-specific variability. According to Bahram et al. (2018), soils exposed to various climatic and edaphic conditions exhibit significant heterogeneity due to environmental filtering on microbial diversity and functional structure at global scales. Because algorithms trained on limited datasets often capture region-specific microbial signatures rather than generally maintained ecological interactions, this ecological variability presents a substantial barrier for AI models. Under real-world field situations, AI models built from constrained temporal or spatial sampling frameworks may see significant drops in predictive ability. Large multilocation datasets, longitudinal sampling techniques, standardized metadata collection, and validation frameworks created specially to evaluate ecological transferability across diverse agricultural contexts are, therefore, necessary to address site-specific heterogeneity.

### 8.5 Regulatory supervision for machine learning-based biofertilizers

As AI-based microbial design advances, safety evaluations and environmental risk analysis methodologies must also adapt. Singh et al. (2025) pointed out that because of uncertainty about ecological stability and long-term environmental interactions, AI-predicted microbial consortia need considerable field-scale validation prior to agricultural deployment. Standardized criteria for assessing the long-term ecological effects, safety, and environmental impact of AI-designed microbial inoculants are dramatically lacking. Reproducibility and cross-site applicability are affected by the absence of defined frameworks for integrating biological data with digital agriculture systems. Adoption may be further hampered by issues with data ownership, privacy, and governance. Both commercial adoption and environmental safety are at risk due to this regulatory ambiguity. As a result, even though smart agriculture integration presents a great deal of promise for flexible, data-driven management, its widespread implementation necessitates solid, affordable, and interoperable system designs backed by well-defined operational and regulatory frameworks. To guarantee responsible deployment and foster stakeholder trust in AI-enabled agricultural technologies, clear governance frameworks, risk assessment procedures, and validation criteria are required.

## 9 Future roadmap and proposed translational framework

For effective implementation of AI in soil microbiome research, several significant improvements are needed. To capture heterogeneity in climates, soil types, and management approaches, large, well-coordinated datasets from diverse agricultural locations should be compiled. Second, standardized techniques like cross-regional benchmarking, temporal validation, and the use of independent external datasets, in addition to repeated testing using traditional methods, are needed to strengthen model validation. Furthermore, combining microbiome analytics with IoT-based tools, like remote sensing and real-time soil sensors, can facilitate data-driven, dynamic decision-making. Lastly, integrating machine learning with basic biological models can enhance interpretability and resilience; nevertheless, prior to widespread implementation, these hybrid systems need to be evaluated through carefully planned field trials.

**Figure 2:**
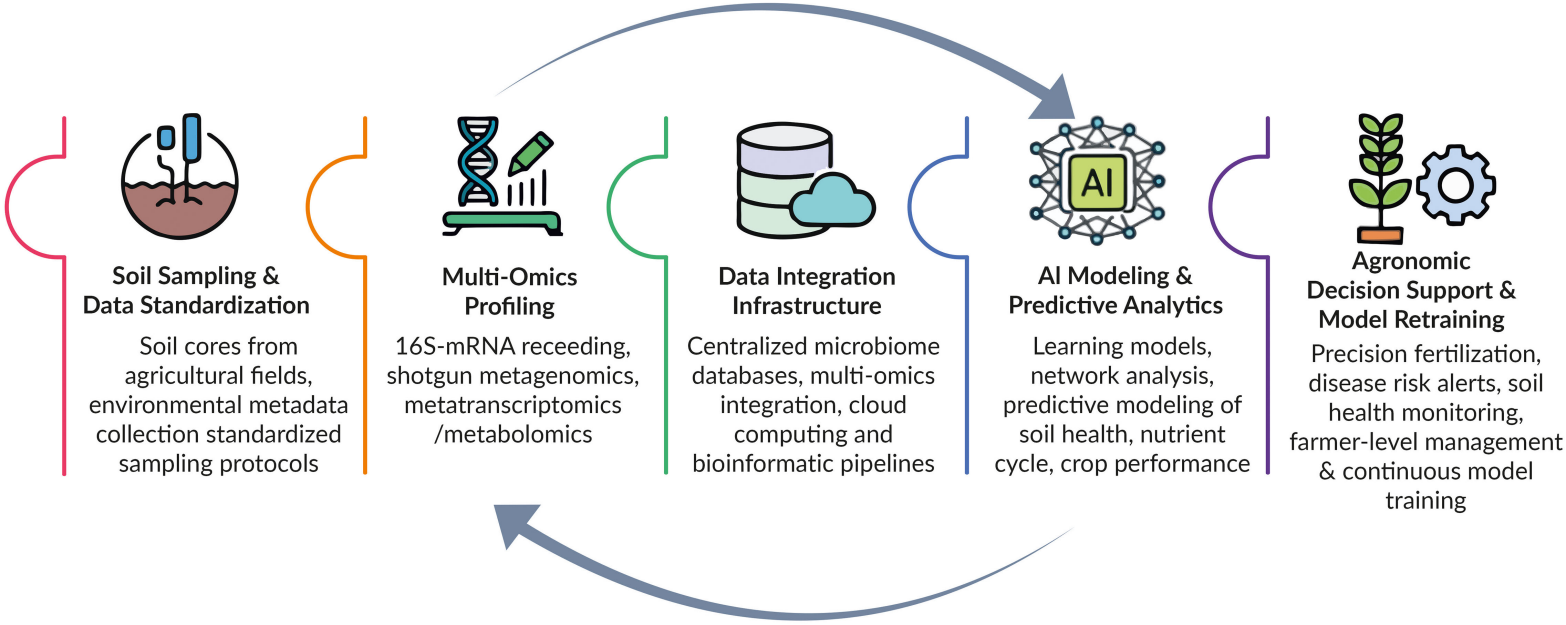
A hypothetical multistage architecture for combining soil microbiome multi-omics data with AI-driven analytical processes and precision agriculture decision support systems. Data collection, multi-omics integration, AI-based modeling, predictive interpretation, and precision agricultural applications with continuous model training are important elements.

Hence, a five-stage implementation framework is suggested to close the gap between agronomic application and computational research (**Figure 2**). Standardized data collection, centralized data integration, AI-driven analytics, agronomic decision assistance, and adaptive model retraining are all integrated into this system. These elements work together to create a closed-loop system that can translate microbiome discoveries into practical management techniques for sustainable agriculture.

### 9.1 Data standardization

Standardized data collection and repeatable sampling techniques are the first steps toward reliable AI modeling in soil microbiome research. Variations in sampling depth, extraction procedures, sequencing platforms, and environmental metadata collection have a significant impact on soil microbial databases. Technical biases introduced by inconsistent procedures might impede cross-study comparability and restrict the creation of reliable predictive models. Therefore, standardized sampling procedures and harmonized metadata are crucial for producing globally comparable microbiological databases. According to Knight et al. (2018), a standardized microbiome workflow can significantly increase reproducibility, lessen technical bias, and boost the dependability of downstream AI-driven predictive analytics. For example, in a hypothetical multilocation maize production system, uniform protocols for sampling depth, DNA extraction, and environmental metadata recording could be used to gather standardized soil samples from geographically separate farms during pre-sowing, vegetative, and reproductive growth stages. Soil physicochemical measures, UAV-derived vegetation indices, climate records, and Internet of Things-based moisture sensing might then be merged with amplicon sequencing in conjunction with targeted nitrogen-cycling and stress-response gene panels. To evaluate ecological transferability across agroecosystems, machine-learning models could be trained utilizing cross-site validation frameworks like leave-one-site-out validation after centralized preprocessing and feature harmonization. Predictive outputs may then produce field-specific recommendations for microbiome-based inoculant deployment, disease-risk management, irrigation timing, and fertilizer optimization. For seasonal model retraining and adaptive recalibration, observed agronomic results such as crop yield, nutrient-use efficiency, disease incidence, and soil health indicators might be reintegrated into the centralized database at the conclusion of each growing season. In addition to providing scalable microbiome-informed decision support for precision agriculture, such iterative closed-loop systems may enhance long-term prediction stability.

### 9.2 Multi-omics profiling

Multi-omics profiling refers to the combined application of genomes, metagenomics, metatranscriptomics, metaproteomics, and metabolomics to provide thorough molecular fingerprints of soil microbial communities in the context of AI in soil microbiome research. Following data standardization and harmonization, multi-omics datasets undergo omics-specific preprocessing, normalization, and functional annotation to ensure biologically meaningful integration and accurate AI-based interpretation. A general normalization technique is inappropriate for microbiome bioinformatics since different omics platforms provide data with varied statistical distributions, sequencing depths, sparsity levels, and technical biases (Knight et al., 2018). Rarefaction, relative abundance scaling, or centered log-ratio (CLR) transformation is frequently used to normalize amplicon sequencing datasets in order to solve compositionality problems. While proteomic and metabolomic datasets usually need median normalization, log transformation, Pareto scaling, or quantile normalization to decrease technical variability and enhance comparability across samples, metagenomic and transcriptomic datasets typically use TPM (transcripts per million) or variance-stabilizing techniques like DESeq2 normalization (Knight et al., 2018; Argelaguet et al., 2020).

Following preprocessing, microbial metabolic functions, nutrient cycling pathways, stress-responsive metabolites, and plant–microbe interaction characteristics are identified using functional and metabolic profiling. Enzyme annotation, metabolite mapping, and pathway reconstruction all heavily rely on databases like KEGG, MetaCyc, and HMDB (Argelaguet et al., 2020). Key metabolic pathways related to nitrogen fixation, phosphate solubilization, carbon sequestration, phytohormone synthesis, and abiotic stress response within soil microbiomes can be identified by utilizing these investigations.

Prior to integration, feature engineering and dimensionality reduction techniques like principal component analysis (PCA), partial least squares discriminant analysis (PLS-DA), autoencoders, and variance filtering can be used to lower the overfitting risks related to high-dimensional omics datasets. Normalized multi-omics datasets can be represented as sample-by-feature matrices after dimensionality reduction and feature engineering, with rows denoting individual soil samples and columns denoting biological variables like taxa, genes, transcripts, proteins, enzymes, or metabolites. Either early integration or late integration techniques can then be used to integrate these matrices into ML and AI workflows (Misra et al., 2019; Argelaguet et al., 2020). Early integration techniques allow for the simultaneous investigation of cross-omics interactions by concatenating data from several omics layers into a single integrated feature matrix before model training. But when the number of predictors significantly exceeds the sample size, this approach can significantly increase dimensionality, sparsity, and feature redundancy, raising the risk of overfitting and unstable model performance. Therefore, with high-dimensional integrated datasets, feature filtering, dimensionality reduction, and rigorous validation become particularly crucial (Misra et al., 2019). On the other hand, late integration techniques integrate predictions or learned representations at later stages of analysis after training distinct models for each omics modality. Although it may restrict direct modeling of cross-omics interactions, this approach may lessen dimensionality-related instability and enable modality-specific optimization.

However, the practical implementation of AI-assisted soil microbiome analytics in agriculture is strongly constrained by the cost, sequencing depth, and computational demands associated with different omics strategies. Currently available evidence shows that simpler methods, like 16S rRNA amplicon sequencing, can frequently achieve predictive accuracies comparable to deeper metagenomic workflows, suggesting that additional omics depth should only be justified when it offers significant improvements in prediction accuracy relative to cost and infrastructure demands (Leite et al., 2022; Kumar et al., 2024). Therefore, targeted microbial marker panels concentrating on functionally significant taxa or genes linked to nutrient cycling, stress resilience, or disease suppression may offer a more practical alternative for routine agricultural monitoring in order to improve scalability rather than solely depending on expensive techniques like shotgun metagenomics or multi-omics integration. In a similar vein, tiered sampling frameworks might be put into place, whereby selective high-resolution metagenomic analysis is only employed for priority or anomalous samples after low-cost amplicon sequencing and soil physicochemical analysis used for broad field screening. We further propose the integration of AI-based feature selection methods to reduce data dimensionality by identifying a limited subset of highly informative microbial indicators, thereby lowering computational requirements without substantially compromising predictive performance.

### 9.3 Data integration and infrastructure

Microbiome datasets should ideally be incorporated into centralized computing infrastructures that can handle large-scale biological, environmental, geographical, and agronomic datasets after standardized data collection and multi-omics profiling. Degnan et al. (2025) showed that by facilitating harmonized analysis across independent cohorts and environmental circumstances, integrated microbiome databases can enhance reproducibility and comparative benchmarking. Sequencing outputs, environmental measurements, geographic data, climatic variables, and agronomic management metadata are often combined in heterogeneous datasets produced by soil microbiome studies, necessitating scalable computational infrastructure and repeatable bioinformatics workflows for successful integration. Standardized and version-controlled analytical pipelines, which include clear reporting of program versions, parameter settings, reference database versions, and preprocessing operations, are widely advised to increase reproducibility and cross-study comparability. By enabling portable and consistent computational environments across platforms, containerization frameworks like Docker or singularity may further improve repeatability. For microbiome, environmental, and agronomic datasets to be interoperably integrated across repositories and research groups, harmonized metadata standards and established ontologies are also crucial. Consistent data storage, cross-regional comparisons, metadata harmonization, and the conversion of raw sequencing reads into repeatable feature tables like amplicon sequence variants (ASVs) or operational taxonomic units (OTUs) are made possible by centralized microbiome repositories and shared computational platforms. Transparency, repeatability, and independent benchmarking of AI-based predictive frameworks in soil microbiome research can be further strengthened by making sequencing datasets, processed feature matrices, metadata, and analysis code publicly available.

### 9.4 AI modeling and predictive analytics

AI and ML algorithms are now employed to understand intricate non-linear correlations between soil microbiome composition, environmental factors, plant characteristics, and agricultural outcomes after multi-omics integration. RF, SVM, gradient boosting techniques, artificial neural networks (ANNs), and deep learning frameworks are often used models for regression and predictive classification applications. Predictions about crop yield, nutrient cycling, disease suppression, soil fertility dynamics, and environmental stress responses can all be supported by these methods (Pace et al., 2025; Walsh et al., 2024). These models are usually trained on standardized datasets that allow for the prediction of microbial and ecosystem responses under various environmental conditions by integrating taxonomic, functional, and metabolic information with soil physicochemical characteristics, climatic variables, and management practices. For precision agriculture and ecosystem management, AI thus converts descriptive soil microbiome statistics into proactive decision support systems.

AI-based models should be compared to traditional statistical and ecological modeling techniques, such as linear regression models, mixed-effects models, abiotic-only models, soil–climate baseline models, and microbiome-only predictive frameworks, to provide methodological robustness and biological relevance. Determining if microbiome-derived features considerably enhance prediction performance beyond traditional environmental predictors alone requires comparative benchmarking. Therefore, a variety of performance indicators, such as accuracy, precision, recall, F1 score, area under the receiver operating characteristic curve (AUC-ROC), and cross-validation stability, should be included in model evaluation.

Furthermore, predictions for new and unseen field sites must be rigorously validated using fully independent external datasets gathered across various locations, climatic zones, soil types, and management practices for applications involving geographically distinct or previously uncharacterized agricultural systems. For widespread deployment or translational implementation, internal cross-validation or random train-test partitioning alone might not be sufficient to capture ecological heterogeneity (Asnicar et al., 2024; Walsh et al., 2024). Therefore, independent site-level validation is especially crucial for assessing model generalizability, transferability, and real-world agricultural applicability.

Dynamic modeling of seasonal microbiome shifts, temporal nutrient fluctuations, and longitudinal soil-health trajectories may be further supported by recurrent neural networks (RNNs), long short-term memory (LSTM) architectures, and similar temporal deep learning techniques. To prevent overfitting and erroneous extrapolation, these models need sufficiently large longitudinal datasets with repeated temporal observations and independent validation (Przymus et al., 2025). Therefore, the use of RNN- or LSTM-based frameworks for predictive agricultural decision-making should be regarded as exploratory rather than completely operational in the absence of reliable time-series datasets.

### 9.5 Agronomic decisions support and field implementation

Translating prediction results into practical agronomic decision support tools for field-level application is an important phase of the AI-enabled soil microbiome framework. As observed through literature, integrated AI and multi-omics models can help optimize fertilizer application, irrigation scheduling, crop rotation strategies, disease management, soil restoration techniques, and precision microbiome-based interventions to increase agricultural productivity and sustainability. These systems may provide site-specific and data-driven agricultural management recommendations by combining microbiome-derived indicators with soil physicochemical characteristics, meteorological variables, and crop performance data. However, clearly defined decision criteria, open communication of forecast uncertainty, economic cost–benefit analysis, regulatory compliance, and usability in real-world field settings are all necessary for effective agricultural deployment. Therefore, decision support platforms should prioritize model interpretability, scalability, and practical usability to ensure successful adoption by farmers, agronomists, and agricultural stakeholders. Explainable AI (XAI) techniques, like SHAP (SHapley Additive exPlanations) and feature-importance analyses, are known to enhance transparency by identifying the microbial, environmental, and agronomic variables that majorly influence predictive outcomes (Asnicar et al., 2024). Such interpretability is particularly important for improving user trust, supporting regulatory acceptance, and facilitating biologically informed decision-making in precision agriculture systems.

Thorough validation under actual agricultural conditions across a variety of geographical regions, cropping systems, soil types, and climatic variables is necessary for successful field application. Because of environmental heterogeneity, management variations, and temporal oscillations in microbial community dynamics, AI-driven recommendations developed in controlled experimental settings could not necessarily translate to varied field environments. Thus, to sustain agronomic relevance and forecast reliability over time, ongoing model recalibration, longitudinal monitoring, and adaptive learning techniques are required.

Real-time monitoring and precise decision-making capabilities could be further improved by integrating AI-driven microbiome analytics with IoT sensors, remote sensing platforms, geographic information systems (GIS), and digital agriculture technologies (Javaid et al., 2022). Nevertheless, issues regarding data interoperability, computational infrastructure, economic viability, farmer accessibility, and uniform regulatory frameworks must still be addressed before large-scale translational deployment can be achieved.

## 10 Conclusion and future perspectives

Artificial intelligence (AI) continues to show the potential for transforming soil microbiome-driven agriculture from a descriptive research field into a predictive and translational platform for precision and sustainable farming. However, despite substantial advances in sequencing and machine learning technologies, data heterogeneity, lack of standardized procedures, longitudinal datasets, ecological variability, and limited external validation across many agroecosystems continue to hinder practical adoption.

This study emphasizes the need for biologically interpretable, standardized, and field-validated analytical frameworks in addition to sophisticated computational models for the effective integration of AI with soil microbiome research. Importantly, a translational implementation framework is proposed, integrating multi-omics profiling, standardized data preprocessing, robust AI benchmarking, external validation, and agronomic decision support systems to improve the reliability and real-world applicability of microbiome-informed agricultural interventions. It may, therefore, provide actionable guidance for researchers, policymakers, and agricultural stakeholders seeking to translate AI-enabled soil microbiome insights into practical, economically viable, and scalable farming solutions.

Future research needs to prioritize explainable AI, independent validation of field sites, longitudinal monitoring of ecosystems, and scalable digital agriculture platforms that can work under heterogeneous environmental conditions. Rather than replacing ecological understanding, AI should serve as a translational bridge across microbial ecology, systems biology, and precision agriculture to develop resilient, climate-smart and sustainability-oriented farming systems. Such a transition may ultimately redefine the future of precision agriculture by integrating ecological functionality, computational intelligence, and translational applicability into a unified framework for global food and environmental security.
